# Development of a Digital Patient Education Tool for Patients With Cancer During the COVID-19 Pandemic

**DOI:** 10.2196/23637

**Published:** 2021-06-21

**Authors:** Sena Turkdogan, Gabriel Schnitman, Tianci Wang, Raphael Gotlieb, Jeffrey How, Walter Henri Gotlieb

**Affiliations:** 1 Department of Otolaryngology - Head and Neck Surgery McGill University Montreal, QC Canada; 2 Department of Experimental Surgery McGill University Montreal, QC Canada; 3 Department of Physiology McGill University Montreal, QC Canada; 4 Precare Inc Montreal, QC Canada; 5 Department of Gynecologic Oncology and Reproductive Medicine MD Anderson Cancer Center Houston, TX United States; 6 Division of Gynecologic Oncology Jewish General Hospital Montreal, QC Canada

**Keywords:** digital health, eHealth, patient education, COVID-19

## Abstract

**Background:**

Due to the COVID-19 pandemic, a large portion of oncology consultations have been conducted remotely. The maladaptation or compromise of care could negatively impact oncology patients and their disease management.

**Objective:**

We aimed to describe the development and implementation process of a web-based, animated patient education tool that supports oncology patients remotely in the context of fewer in-person interactions with health care providers.

**Methods:**

The platform created presents multilingual oncology care instructions. Animations concerning cancer care and mental health during the COVID-19 pandemic as well as immunotherapy and chemotherapy guides were the major areas of focus and represented 6 final produced video guides.

**Results:**

The videos were watched 1244 times in a period of 6 months. The most watched animation was the COVID-19 & Oncology guide (viewed 565 times), followed by the video concerning general treatment orientations (viewed 249 times) and the video titled “Chemotherapy” (viewed 205 times). Although viewers were equally distributed among the age groups, most were aged 25 to 34 years (342/1244, 27.5%) and were females (745/1244, 59.9%).

**Conclusions:**

The implementation of a patient education platform can be designed to prepare patients and their caregivers for their treatment and thus improve outcomes and satisfaction by using a methodical and collaborative approach. Multimedia tools allow a portion of a patient’s care to occur in a home setting, thereby freeing them from the need for hospital resources.

## Introduction

Over the past few months, the drastic escalation of the COVID-19 pandemic has imposed unprecedented challenges to the global health care system. Oncology patients are among the most vulnerable populations. Compared to patients without cancer, oncology patients are at a higher risk of contracting (18% vs 0.29%), as well as developing (39% vs. 8%), severe complications of COVID-19 [1,2]. Liu et al [3] recently examined the use of telehealth in oncology during the pandemic and discussed health care services that can be provided through digital means. Indeed, telemedicine visits have been rapidly adopted to prevent disease transmission, and the uptake of digital tools that facilitate remote networking has increased significantly [4,5]. However, despite the increasing value of telehealth and the development of vast educational resources, little has been implemented to address demands from the patients’ perspective [6,7]. Most patient education platforms that are available attempt to explain, in simple terms, the technical and medical aspects of a certain condition while limiting the information regarding what patients wish to learn, such as information about their treatment or recovery process. Additionally, these resources have not been properly studied by academia and face difficulty in penetrating large scales of usage and feedback.

The development of digital education tools for oncology patients could fill an unmet need in the growing telehealth environment, thereby empowering patients and reducing anxiety [6]. An ideal patient education tool that addresses this demand should function in support of care by medical professionals and provide knowledge and instructions tailored to oncology patients during the COVID-19 pandemic. Additionally, the material presented should be based on updated medical information and be readily accessible and understandable.

The COVID-19 crisis has emphasized the demand for an organized, evidence-based, digital medical education tool that can satisfy patients’ needs for knowledge. Although health information has become readily available on the internet, they are often ill adapted to the population’s health literacy [8]. Furthermore, the lack of screening of published materials results in a heightened risk of misinformation [9]. Numerous studies have underlined the value of eHealth in the context of the pandemic and have provided evidence of the effectiveness of digital tools [7].

We propose an approach to the development of video guides for a web-based education platform for oncology patients that ensures that patients are equipped with adequate and accurate medical information. Similar strategies have been explored for the management of other diseases during the COVID-19 pandemic, such as the use of a storyboard-style, web-based education tool for patients undergoing otolaryngologic surgery [10]. The implementation of an oncology patient education tool that allows patients to provide feedback and communicate digitally could contribute toward overcoming challenges during this emotionally taxing period by alleviating anxiety and confusion, especially those among newly diagnosed oncology patients.

## Methods

This is a descriptive study on the development process of a tool for addressing oncology patients’ needs for educational information during the COVID-19 pandemic. The creation of this tool was conducted with a web-based platform via a collaborative process that united efforts from university, industry, and hospital departments. The platform was created to serve as an engaging access portal for patients to intuitively navigate information.

This study was conducted from April to August 2020, that is, after the pandemic became a well-established global threat that forced health care institutions to deliver patient education via novel procedural methods.

This study’s procedures were performed in 4 major steps. The first step was topic selection. The topics covered in the education materials sought to directly target oncology patients’ informational needs during the COVID-19 pandemic. Patients’ informational needs were identified by consulting a cancer network consisting of patient advocates, nurses, physicians, and other health care professionals. Patients’ opinions were also obtained either via one-on-one, in-person conversations or from written texts that were submitted to an anonymous, nonstandardized suggestion box located in the oncology center.

The second step was content development. Initially, a broad literature review was performed on medical databases such as UpToDate and PubMed. The topics searched were oncology and COVID-19, and this led to a list of determined topics to be mentioned. A summary of all relevant information was created, and a main document containing the content was developed. Afterward, this information was adapted into a video script and analyzed for language and cultural adjustments according to the target population.

The third step was video production. To aid patients’ comprehension and connect with a diverse population, the delivery method chosen was animated videos with audio voice-overs that were spoken clearly. The videos were created by professionals via animation software and offered on our web-based platform [11]*.* Audio and text were presented in English and French, and subtitles in 20 languages were offered to accommodate for the various cultural backgrounds of the patient population.

The fourth step was implementation and feedback gathering. Implementation was carried out on the web-based platform and presented to patients by the oncology health care teams during consultations. Resources were explained to patients during their clinical visit in the same way as when they would be handed an information pamphlet. Patients were able to access the platform at any time, and feedback was obtained in order to identify patient needs. Patients were able to ask questions, communicate their concerns, and provide feedback directly on the platform or during their consultations. Multiple iterations were carried out based on feedback from health care professionals and patient representatives. Data analytics was performed, which allowed for assessments of acquisition, conversion, and behavior. These analytical data sets provided constant suggestions for improvements to find the balance between the standardization of content and the personalization of educational experiences to individual needs.

All data used were anonymous and summarized in a password-protected, web-based database. The data analysis was performed using Microsoft Office Excel software (licensed version 16.36).

## Results

The creation of well-balanced, evidence-based patient education videos required a multidisciplinary team from different branches of health care and digital media that encompassed professionals such as physicians, nurses, psychologists, social workers, and graphic designers. This approach ensured the accuracy, validity, and efficiency of the medical information included in the script.

Based on the information obtained from health care professionals and oncology patients, the selected topics for the videos were related to cancer treatment options, good practices of self-care, and disease prevention for oncology patients during the COVID-19 pandemic. Animations concerning cancer care and mental health during the COVID-19 pandemic as well as immunotherapy and chemotherapy guides were the major areas of focus and represented 6 final produced video guides. A sample video guide that was developed during this process as well as 2 screenshots can be found in Multimedia Appendices 1 and 2.

The first guide addressed hospital treatments for oncology patients that have been maintained during the pandemic, which was the biggest demand from patients. The aim was to demonstrate treatment options for cancer care and procedures for hospital attendance and to provide general follow-up information. The second guide contained a general overview of the COVID-19 pandemic, the disease, its symptoms, general preventive measures, and treatment perspectives. The third guide was developed to address essential information on infection prevention and alert signs for oncology patients during the pandemic. Some of the topics mentioned in this video were related to SARS-CoV-2, handwashing techniques, symptoms, and complications. The fourth guide addressed the mental health consequences of the social isolation resulting from the pandemic. It presented a brief contextualization of the reasons behind psychological distress and techniques for improving mental well-being during home care for oncology patients. The fifth and sixth guides were related to oncology treatments (namely, immunotherapy and chemotherapy) that may still be occurring during the social isolation period and therefore require specific attention.

The length of each guide varied from 6 minutes (video: “COVID-19 for Oncology”) to 13.5 minutes (video: “Chemotherapy”). In order to create a welcoming viewing environment for patients, scenarios for the animations were created to represent either health care institutions or a house, depending on the context of the content being presented. The videos were watched 1244 times in a period of 6 months. The most watched animation was the COVID-19 & Oncology guide (viewed 565 times), followed by the video concerning general treatment orientations (viewed 249 times) and the video titled “Chemotherapy” (viewed 205 times). Although viewers were equally distributed among the age groups, most viewers were aged 25 to 34 years (342/1244, 27.5%). Most participants were female (745/1244, 59.9%). Demographic data are shown in Table 1.

**Table 1:** Demographic data (N=1244).

Characteristics			Viewers, n (%)
**Sex**			
	Male	499 (40.1)	
	Female	745 (59.9)	
**Age (years)**			
	18-24	153 (12.3)	
	25-34	342 (27.5)	
	35-44	214 (17.2)	
	45-54	193 (15.5)	
	55-64	174 (14)	
	>65	168 (13.5)	

## Discussion

### Principal Findings

In the field of oncology, digital patient education is relatively new and lacks an organized strategy. Nonetheless, research studies have provided promising insights. A study conducted by Sun et al [12] assessed the effectiveness of a multimedia self-management intervention for patients with lung cancer, which consisted of a video, a handbook, and phone calls for discussing disease pathophysiology and recovery care. Significantly improved postoperative emotional quality of life scores were reported from the intervention group; upward trends in the assessments of self-efficacy and surgery-related knowledge were also reported [12].

Digital media for patient education offers various advantages over traditional media (eg, pamphlets or handouts), as depicted in Table 2. Among the various digital tools, the use of multimedia or videos has been shown to be more effective than the use of pure texts [8,13]. Walker and Podbilewicz-Schuller [13] reported that patients with breast cancer who received videotaped education prior to their consultations reported higher satisfaction and reduced stress levels as well as better preparedness when asking questions during the consultations compared to those who received information booklets. Among the different formats of videos available, animations possess the advantage of illustrating complex materials in a vivid way to facilitate understanding. When combined with the guidance of spoken texts, animations may boost people’s ability to process information by simultaneously exciting their audio and visual receptive channels [14,15]. A recent study demonstrated that presenting health information through an animation combined with audible text, when compared with using illustrations or written texts, results in a significantly higher recall rate, especially among those with lower health literacy levels [8].

**Table 2:** Comparison among different media of patient education.

Characteristics	Digital education	Traditional education	Ideal education for patients with cancer
Accessibility	Free home access	Hospital-dependent access	Easy home access
Content	Flexible and adaptable	Fixed	Reliable
Circulation	On the internet globally	Local distribution	Personal devices
Cost	High implementation cost and low maintenance cost	High implementation cost and high maintenance cost	Free of charge

We intended for our tool and the proposed approach to the development process to maintain a high fidelity to evidence-based information while attempting to adapt them to a more engaging medium and effective platform that accommodated content that was in line with the cognitive theory of multimedia learning (CTML). An important aspect of adapting the CTML to patient education is reviewing language content to limit medical jargon and ensure that it is written at a comprehension level that accommodates different health literacy levels [16]. Studies have shown that patients understand medical information better when it is provided (ie, spoken) at a conversational pace and individual speed control is available, when simple words are used, and when a restricted amount of information is presented [14-16]. Additionally, while it is acceptable for medical videos to be as long as needed to provide enough time for vital information to be thoroughly discussed, people should also be considerate of the attention spans of a diverse audience. Detailed or complicated medical guides could be divided into several chapters to promote better engagement and retention.

Another important aspect to consider when developing a web-based medical education platform is its accessibility. Our proposed web-based platform could be accessed for free and without temporal or spatial limits, which provided patients with a channel for central, authoritative sources of medical information at home. Although this may present a financial challenge for the development of any innovation, we believe that this is crucial for achieving the conduction of adequate educational processes. To optimize support for patients with cancer during the COVID-19 pandemic, developing an accessible platform was an imperative action for health care institutions. Collaborations with the health care professionals provided patients with an introduction to the platform and allowed us to validate the platform. As such, these collaborations represented an important link to the implementation process. Partnerships between industry representatives, universities, and health care institutions were established to produce this multifaceted and integrative platform.

The acquisition of feedback from patients and health care professionals for regular evaluation and improvement was also an important aspect of our proposed tool. Through a web-based platform, we were able to obtain regular data and feedback, which allowed for modifications to be made constantly according to patients’ demands. An example of feedback that generated modifications was the fact that we were able to analyze the average age of the viewers that was reported by the platform, which was different from what we expected. This information, in turn, allowed us to adapt the characters shown in the videos. Such data can also serve as indicators for making possible changes in language, style, or scenarios. This adaptability is an important advantage of using animated guides that is not present in live-action videos or printed materials.

Finally, attention to viewers’ diversity in terms of cultural and linguistic backgrounds is valuable in effective communication, which in turn increases viewers’ engagement and comprehension [17]. This effect may additionally be magnified for target populations such as individuals with hearing impairments, individuals with low literacy levels, or minority language speakers [18,19]. Multimedia education may aid in this context by increasing oncology patients’ participation in the healing process, which is vital for better outcomes, as it improves self-care, decision making, and the overall understanding of diseases and treatments [7]. Our proposed platform addresses this diversity and maintains medical accuracy by offering animated video guides in multiple languages and the possibility of individualizing the content to target certain demographics.

This study presents limitations that are intrinsic to the methodological approach and to the implementation process. Patients were not randomized, and intervention outcomes were not quantitatively measured. The results were provided based on the descriptive analysis of the intervention, and no statistical inference testing was applicable. Future studies should pursue analyzing factors that influence oncology patients’ engagement with digital education resources as well as outcome measures, such as medication adherence, the number of hospital visits, and pain control.

### Conclusion

The implementation of an animated patient education platform can be designed to prepare patients and their caregivers for their treatment in an attempt to improve outcomes and satisfaction, by using a methodical and collaborative approach. Multimedia tools allow a portion of a patient’s care to occur in a home setting, thereby freeing them from the need for hospital resources. During the pandemic, the rapid adoption of web-based care might not be sufficient to cover a patient's oncology and emotional needs. We describe the framework for producing and implementing web-based animations that serve as educational tools for oncology patients and their personal support networks.

## Appendix

Multimedia Appendix 1 Still images of animated videos.

Multimedia Appendix 2 Sample video: oncology care during the COVID-19 pandemic.

## Abbreviations

CTML: cognitive theory of multimedia learning
