# Supplementary figures and images for: Development of a Digital Patient Education Tool for Patients With Cancer During the COVID-19 Pandemic
Source: JMIR Cancer. 2021 Jun 21;7(2):e23637. doi: 10.2196/23637 (PMC8218900; doi:10.2196/23637)

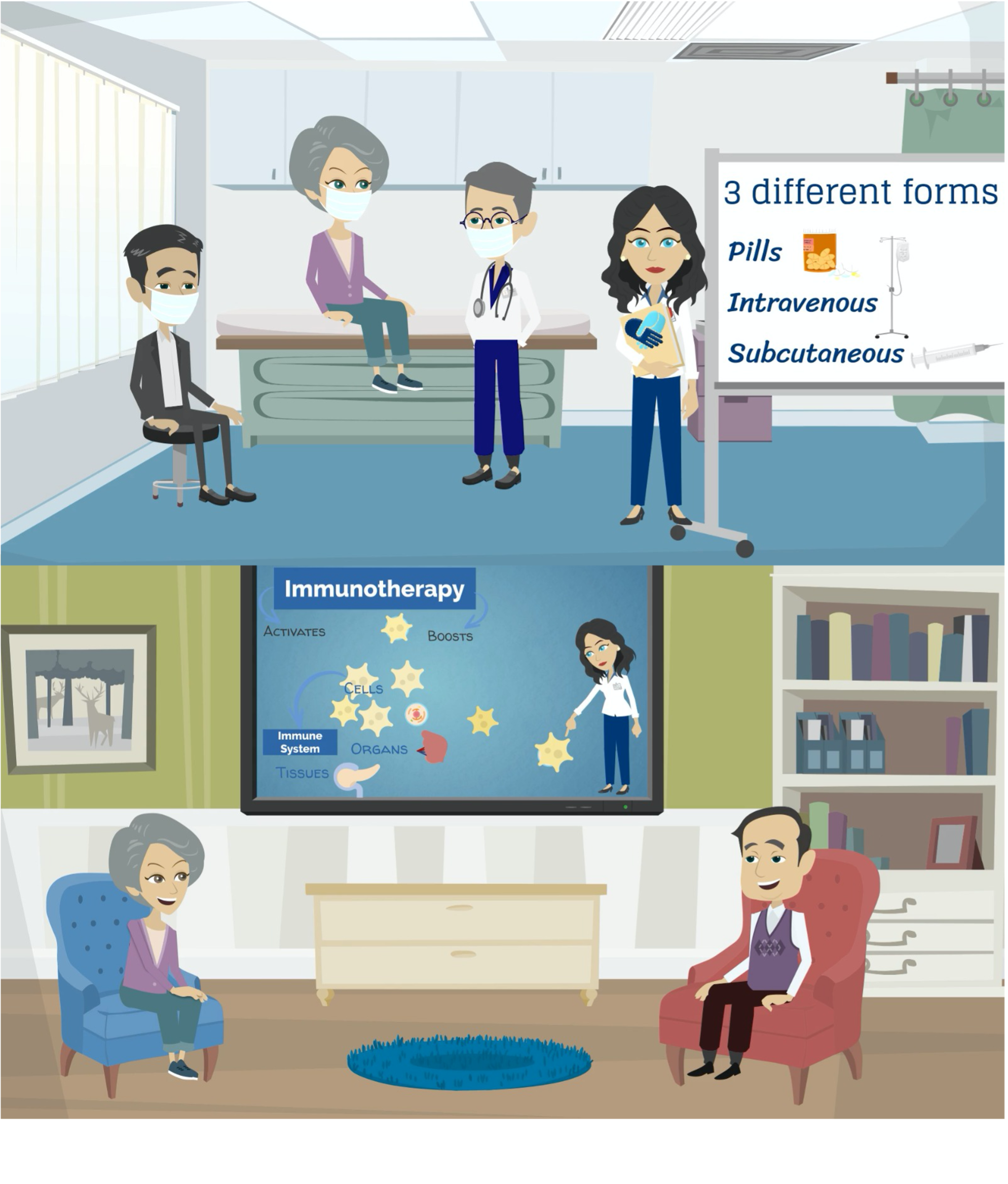

Supplement: Multimedia Appendix 1 [file cancer_v7i2e23637_app1.png]
